# Morbidity and mortality related to type II odontoid fractures in octogenarians undergoing surgery: a retrospective study with 5 year follow up

**DOI:** 10.3389/fmed.2023.1082848

**Published:** 2023-09-29

**Authors:** Pavlina Lenga, Gelo Gülec, Karl Kiening, Andreas W. Unterberg, Basem Ishak

**Affiliations:** Department of Neurosurgery, Heidelberg University Hospital, Heidelberg, Germany

**Keywords:** odontoid fractures, low energy trauma, octogenarians, fusion, comorbidities

## Abstract

**Introduction:**

The prevalence of trauma is increasing in the geriatric population. The optimal therapy for type II odontoid fractures in the elderly is controversial. This study aims to assess the morbidity and mortality associated with odontoid fractures in octogenarians undergoing C1/C2 posterior screw fixation and describe the perioperative and post-operative complications and risk factors associated with mortality.

**Materials and methods:**

Electronic medical records from a single institution pertaining to the period between September 2005 and December 2020 were retrieved. Data on patient demographics, neurological conditions, surgical characteristics, complications, hospital course, and 90-day mortality were collected.

**Results:**

Over a 16-year period, 60 patients aged ≥80 years diagnosed with type II odontoid fractures were enrolled in the study. The mean age was 85.0 ± 1.9 years. The mean Charlson Comorbidity Index (CCI) was >6 indicating a poor baseline reserve (8.5 ± 1.9), while cardiovascular diseases were the most prevalent among comorbidities. The mean surgical duration was 217.5 ± 65.9 min, with a mean blood loss of 725.5 ± 275.7 mL. The in-hospital was 5–0% and the 90-day mortality rates increased at 10.0%. No revision surgery was needed in any of the cases. Intraoperative and post-operative X-ray and computed tomography (CT) imaging revealed correct screw placement. Proper alignment of the atlantoaxial spine and fusion could be achieved in all cases. The unique risk factors for mortality included the presence of comorbidities and the occurrence of post-operative complications.

**Conclusion:**

The complication and mortality rates associated with odontoid fractures in octogenarians are relatively high. However, the therapeutic goals in this population also include bone union and preservation of neurological status. Despite the often-high comorbidity rate, we still recommend that surgery should be considered in patients over 80 years. However, it is necessary to evaluate several approaches when treating such frail patients.

## 1. Introduction

With the global trend of increasing life expectancy, healthcare systems worldwide are confronted with the medical needs of older patients. Thus, medical/surgical strategies need to be adjusted to the unique needs of this steadily evolving population. For instance, geriatric trauma continues to increase in prevalence, and it is predicted that by the year 2050, nearly 40% of all injured patients will be aged >65 years. In terms of spine fractures, odontoid fractures constitute the most common fracture of the axis, and the most prevalent type of fracture of the cervical spine among patients aged 65 years and older (1, 2).

The susceptibility of older patients to such fractures might be attributed to the loss of bone mineral density due to the increased incidence of osteoporosis or to degenerative disorders causing the calcification of ligaments; these conditions result in a decreased ability to absorb traumatic impact. Consequently, a low velocity trauma such as a fall from a standing posture can cause such fractures (3).

In spite of the high prevalence of odontoid fractures among the elderly (range 2.4–4.7%) (4), its treatment remains controversial. Previous studies suggest that conservative management such as halo vest immobilization or rigid cervical orthosis lead to decreased rates of fusion and contribute to higher rates of chronic pain, neurological deterioration, morbidity, and mortality (5, 6). In contrast, surgical management such as C1/C2 posterior screw fixation has been reported to produce promising results with high rates of biomechanical stability and fusion compared to conservative management (7, 8). However, a major drawback of surgical techniques is, due to the poor baseline reserve in the elderly, that the surgical risks are high in this population (9). Striking the right balance between risk and benefit and formulating the right therapeutic strategy remains a difficult proposition.

To address this issue, the present study seeks to describe the morbidity and mortality rates in octogenarians with odontoid fractures undergoing C1/C2 posterior screw fixation and assess the perioperative and post-operative complications and risk factors associated with mortality.

## 2. Materials and methods

### 2.1. Study design and patient characteristics

Clinical and imaging data were retrospectively collected for the period between September 2005 and December 2020 from our institution’s database. This study was approved by the local ethics committee of our institution (approval number 880/2021) and was conducted in accordance with the Declaration of Helsinki. The requirement for informed consent was waived due to the retrospective nature of the study.

**FIGURE 1:**
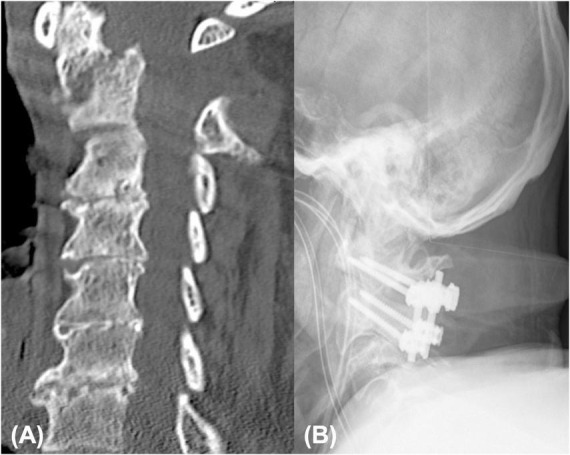
A total of 85 year male patient presenting with arm monoparesis: emergency CT showing type II odontoid fracture with anterior displacement **(A)**, post-operative anterior-posterior radiographs displaying posterior screw fixation of C1/C2 **(B)**.

Patients aged ≥80 years with type II odontoid fractures diagnosed on both cervical spine radiograph and computed tomography (CT) images were included (Figure 1). A CT-angiography was performed to examine the presence of anatomical abnormalities or kinking in the course of the vertebral artery before surgery. Magnetic resonance imaging (MRI) of the cervical spine was performed to evaluate the integrity of the spinal ligaments. The exclusion criteria were as follows: congenital instability, rheumatoid arthritis, instability caused by a tumor, spinal infections, and previous cervical surgery. The German guidelines for trauma mechanisms were used to define low energy trauma (LET) and the patients’ injuries were classified accordingly (10). LET was defined as a fall from a sitting or standing position or a low height (<1 m) (10).

### 2.2. Outcome parameters

Data regarding patient demographics, comorbidities, American Society of Anesthesiologists scores, surgical duration, number of treated spinal levels, perioperative and post-operative complications, length of hospital stay, intensive care unit (ICU) stay, readmission, reoperation, and mortality were retrieved from the patients’ electronic records. Comorbidities present preoperatively were assessed using the age-adjusted Charlson Comorbidity Index (CCI). The CCI was calculated for each patient and classified as no comorbidity, minimal comorbidity, moderate comorbidity, or severe comorbidity (CCI of 0, 1 or 2, 3 to 5, and >5, respectively) (11, 12). The pre-treatment neurological condition was assessed using the American Spinal Injury Association impairment scale (AIS A: complete sensory and motor loss, B: sensory loss incomplete but complete motor loss, C: motor loss incomplete with key muscle test less than a grade of 3; D: motor loss incomplete with key muscle test more than a grade of 3; E: complete return of all motor and sensory function) (13). Post-treatment AIS data were obtained from the last documented clinical encounter. Routine clinical and radiological follow-up examinations were performed before discharge and 3 months after surgery according to our institutional standards. The follow-up period was between 3 and 72 months after surgery. Conventional radiographs in the anteroposterior and lateral views were obtained to evaluate the screw position and fusion rates.

#### 2.2.1. Decision process

Patients aged ≥80 years with an acute unstable odontoid fracture type II were enrolled in the study. The diagnosis of acute odontoid fracture was based on a thin-slice computed tomography (CT) scan. The decision making was jointly guided by both clinical and radiological parameters. In cooperation with experienced neuroradiologists, meticulous assessments of the CT images and following predictors were considered, as previously recommended (14–16): non-union displacement >4 mm, posterior displacement, age of trauma, and fracture coagulation of more than 10°.

### 2.3. Surgical technique

A modified Goel-Harms technique in conjunction with a posterior arch C1 lateral mass screw (PALM) was used for the C1/C2 posterior screw fixation (17, 18). By applying this technique, the risk of bleeding from the epidural venous plexus as well as postsurgical neuralgia resulting from the manipulation of the C2 nerve root can be mitigated as the posterior arch of C1 is used as an entry point. C2 pedicle screws were inserted according to Harms’ technique in most cases (19). In some cases, the C2 screws also were inserted according to Harms’ technique in the pars of C2, lateral to the superior margin of the C2 lamina (19). All instrumented surgeries were performed using a CT-based point-to-point navigation system to maximize safety, as previously described by our study group (20). All instrumented surgeries were performed using a CT-based point-to-point navigation system to maximize safety, as previously described by our study group (20). After surgery, none of the patients were kept in a cervical collar, as recommended by the German Guidelines of the spine community for the management of cervical fractures encompassing treatment approaches for odontoid type II fractures (21).

#### 2.3.1. Post-operative care and monitoring

Following the surgical procedure, all patients were transferred to the Intensive Care Unit (ICU) for close monitoring. This standard protocol was adhered to ensure optimal post-operative care, allowing for the immediate detection and management of any potential complications. Each patient remained in the ICU for a minimum duration of 1 day, with the exact duration determined by the attending physician’s assessment of the patient’s recovery and stability.

### 2.4. Statistical analysis

Categorical variables are presented as numbers and percentages. Continuous variables are presented as means ± standard deviations; the Shapiro–Wilk test was used to verify whether the data distribution was normal. Baseline characteristics, duration of surgery, number of treated spinal levels, perioperative and post-operative complications, length of stay (LOS), ICU stay, readmissions, reoperations, and mortality were compared groupwise using independent *t*-tests for continuous variables and chi-squared tests for categorical variables. The chi square test was used to evaluate changes in neurological status (AIS). In the second-stage analysis, a binary logistic regression analysis was performed to identify the risk factors for mortality. Statistical significance was set at a *p*-value of 0.05 or less.

## 3. Results

### 3.1. Patient demographics and baseline characteristics

Sixty patients aged ≥80 years diagnosed with type II odontoid fractures were enrolled in the study over a 16-year period. The mean age was 85.0 ± 1.9 years; there was a predominance of female patients (*n* = 38, 63.3%) among the study population. The mean CCI was >6, indicating a poor baseline reserve (8.5 ± 1.9). The prevalence of arterial hypertension, coronary heart disease, and atrial fibrillation was high (*n* = 53, 88.3%; *n* = 37, 61.7%; *n* = 27, 45.0%, respectively). The mean MS was 86.9 ± 20.1, indicating the presence of a new motor deficit. A detailed breakdown of the patient characteristics is presented in Table 1.

**TABLE 1:** Baseline characteristics.

Characteristic	Value
Number of patients	60
Age, years (mean, SD)	85.0 (1.9)
**Sex (*n*, %)**	
Male	22 (36.7)
Female	38 (63.3)
Body mass index, kg/m^2^ (mean, SD)	30.8 (5.1)
**Comorbidities**	
Age-adjusted CCI score (mean, SD)	8.5 (1.9)
Arterial hypertension (*n*, %)	53 (88.3)
Myocardial infarction (*n*, %)	24 (40.0)
Coronary heart disease (*n*, %)	37 (61.7)
Atrial fibrillation (*n*, %)	27 (45.0)
Heart failure (*n*, %)	20 (33.3)
COPD (*n*, %)	10 (16.7)
Diabetes mellitus type II (*n*, %)	15 (25.0)
Renal failure (*n*, %)	13 (21.7)
Liver disease (*n*, %)	4 (6.7)
Gastrointestinal ulcer (*n*, %)	14 (23.3)
TIA/stroke (*n*, %)	13 (21.7)
Malignancy (*n*, %)	14 (23.3)
Dementia (*n*, %)	17 (28.3)
Previous spinal surgery (*n*, %)	3 (5.0)
**ASA class (*n*, %)**	
II	16 (26.6)
III	38 (63.3)
IV	5 (8.3)
Preoperative MS (mean, SD)	86.9 (20.1)

ASA, American society of anesthesiologists; CCI, Charlson comorbidity index; COPD, chronic obstructive pulmonary disease; MS, motor score of the American spinal injury association grading system; SD, standard deviation; TIA, transient ischemic attack.

### 3.2. Surgical characteristics

As shown in Table 2, the mean surgical duration was 217.5 ± 65.9 min, with a mean blood loss volume of 725.5 ± 275.7 mL. The mean number of decompressed levels was 1.7 ± 0.8. The mean ICU stay was 3.9 ± 0.3 days, while the hospital stay lasted 13.0 ± 8.5 days. During the hospital stay, three patients (5.0%) died from septic events, while another three (5.0%) died 3 months after surgery due to cardiovascular disease. No further surgeries were required during the follow-up period. The AIS improved significantly after surgery (*p* < 0.001). Overall, the mean follow-up period was 67.3 ± 17.7 months, and no additional surgery to treat secondary instability was necessary. Intraoperative and post-operative radiographic and CT imaging revealed correct screw placement. Proper alignment of the atlantoaxial spine could be achieved in all cases. Fusion could be achieved in all patients by clearly visible, continuous bony trabeculation as evaluated by radiographic imaging at the final follow-up. During the follow up period, three additional patients deceased due to an acute heart failure, while no patients died due to postsurgical complications. The overall mortality rate increased to 15.0% (*n* = 9/60).

**TABLE 2:** Perioperative and post-operative surgical characteristics and clinical course of 60 patients who underwent C1–C2 posterior screw fixation.

Characteristic	Value
Surgical duration, min	217.5 (65.9)
Estimated blood loss, mL	725.5 (275.7)
Blood transfusion (*n*, %)	16 (26.7)
Hospital stay, days	13.0 (8.5)
ICU stay, days	3.9 (0.3)
**Mortality**	
In-hospital (*n*, %)	3 (5.0)
90-day (*n*, %)	3 (5.0)
30-day readmission (*n*, %)	0 (0.0)
Post-operative MS	93.9 (15.9)

Values represent the mean (SD) except where otherwise indicated.

ICU, intensive care unit; MS, motor score of the American spinal injury association grading system; SD, standard deviation.

### 3.3. Occurrence of adverse events and potential risk factors

The most prevalent complications were pneumonia (20.0%), followed by urinary tract infection (8.3%), acute heart failure (6.7%), pleural effusion (6.7%), and septic events (5%). Detailed data regarding post-operative complications are presented in Table 3. In the second stage analysis, we investigated the potential risk factors for mortality. The presence of comorbidities and post-operative complications were unique risk factors for mortality; surgical duration, estimated blood loss, and hospital or ICU stay were not risk factors for mortality (Table 4). Separate analyses were performed to evaluate potential collinearity between variables of interest. When hospital stay and ICU stay, as well as the duration of surgery and blood loss, were individually introduced into the multivariate model, the outcomes remained consistent with the primary results.

**TABLE 3:** Occurrence of adverse events in octogenarian patients (*N* = 60) who underwent decompression surgery.

Event	Number of patients (%)
Deep wound infection	1 (1.7)
Acute heart failure	4 (6.7)
Pulmonary embolism	2 (3.3)
Pneumonia	12 (20.0)
Sepsis	3 (5.0)
Pleural effusion	4 (6.7)
Ileus	2 (3.3)
Urinary tract infection	5 (8.3)
Dysphagia	1 (1.7)

**TABLE 4:** Risk factors associated with mortality.

Risk factor	OR (95% CI)	*p*-value
Age-adjusted CCI score	1.4 (1.1–3.7)	**0.004**
Preoperative MS	0.9 (0.8–1.0)	0.433
Duration of surgery	1.1 (1.0–1.5)	0.590
Estimated blood loss	1.0 (0.9–1.1)	0.696
Length of ICU stay	0.8 (0.6–1.2)	0.308
Length of hospital stay	1.2 (0.8–1.7)	0.055
Complications	2.2 (1.5–3.7)	**0.006**

CCI, Charlson comorbidity index; CI, confidence interval; ICU, intensive care unit; MS, motor score of the American spinal injury association grading system; OR, odds ratio. Bold values indicate statistically significant results.

## 4. Discussion

Cervical spine fractures are associated with high morbidity and mortality rates in the geriatric population (22–24). Odontoid fractures are highly prevalent among the elderly. Due to degenerative and osteoporotic alteration of the dens axis in this population, minor trauma such as a fall from a standing position can cause such fractures. While odontoid fractures are relatively frequent, their therapeutic management is still a subject of debate; both operative and non-operative treatment options are associated with several disadvantages. It is important to note that the existing evidence is predicated upon a small retrospective series and may not help in resolving the dilemma of whether or not to treat an octogenarian patient with an odontoid fracture.

### 4.1. Summary of results

To the best of our knowledge, this is the largest retrospective series examining the clinical course of surgical treatment for odontoid fractures in patients ages 80 years and older. We assessed the mortality and morbidity rates and determined the risk factors for both mortality and loss of ambulation exclusively among octogenarians undergoing C1/C2 posterior screw fixation in less than 72 h after trauma. Our results revealed that octogenarians presented with a poor baseline history, as indicated by a CCI > 6, with cardiovascular diseases being the most prevalent. Both the in-hospital and 90-day mortality rates were at 5.0%. It is important to emphasize that the cause of death was not surgery-related, but due to events unrelated to the surgical procedure. Of note, the neurological condition, as defined by the AIS, improved significantly after surgery. Most importantly, the presence of comorbidities as well as post-operative complications were unique significant predictors of mortality.

### 4.2. Review of literature

#### 4.2.1. Comorbidities, complications, and clinical course

Performing surgical procedures in older patients for cardiac, orthopedic, or vascular diseases is currently widely accepted, while surgery for traumatic fractures such as odontoid fractures is not yet commonly practiced in the same patient cohort (25). Rivzi et al. reported on a retrospective series of 336 patients with odontoid fractures and analyzed the impact of age and comorbidities on surgical decision-making. They found that preexisting major medical comorbidities were associated with increasing age and a higher level of dependency (26). Note worthily, surgery was mainly preferred in patients with low comorbidity rates as well as independent living, as such patients were more compliant to surgery than their older counterparts. Schoenefeld et al. also showed that surgery was associated with reduced mortality in patients aged between 65 and 74 years, while this association diminished with increasing age; this finding was mainly attributed to the poor baseline reserve of the elderly (27). Issa et al. reported on another retrospective series of nonagenarians undergoing C1/C2 posterior screw fixation due to odontoid fractures, and also found high comorbidity rates; in that study, 80% of the patients were found to have a high risk of perioperative complications (28). In the present study, the rates of comorbidities were high, with a mean CCI of 8.5, indicating poor baseline medical conditions. Nevertheless, each patient underwent emergent surgery due to high grades of dislocation of the dens axis as well as progressive neurological deficit. Emphasizing our findings, age alone should not overshadow clinical necessities when determining the surgical intervention’s appropriateness.

It is often debated whether odontoid fractures should be treated surgically due to the related post-operative complications. Charles et al. in their retrospective study on 204 patients with odontoid fractures reported a mortality rate of 12.7% within 1 year, while the rate for patients aged 70 years and older were significantly higher, at 16.7% (29). It is worth noting that the significant predictors of mortality were increasing age and the present of comorbidities, while the treatment choice (surgery vs. conservative) did not significantly impact the outcome (29); these findings are in line with those from the present study. In another review and meta-analysis examining patients with odontoid fractures undergoing surgery, the overall mortality rate was 10.1%; the in-hospital mortality was 6.2% and the 6- and 12-year mortality rates were at 7.4% (9). The study group postulated that increasing age and underlying diseases were the key factors related to post-operative mortality (in agreement with Charles et al.). Overall, they suggested that surgery and posterior screw fixation might be beneficial for geriatric patients in terms of pain relief and occurrence of post-operative adverse events. However, a major limitation of the study is that the study population consisted of individuals aged 65 years and older and long-term follow up data were not available (9); this lowered the significance of their results, and particularly their extrapolation of the findings to octogenarian patient. Similar to the aforementioned studies, Smith et al. performed a retrospective analysis of 32 octogenarians undergoing conservative or surgical management and reported a mortality rate of 12.5% in the surgically treated and of 15% in the conservatively treated cohort, (difference not significant) (7). This study group also stated that patient baseline history and age were associated with mortality, irrespective of treatment type (25). Similarly, in the case of nonagenarians undergoing surgery for odontoid fractures, the mortality rates are associated with preexisting comorbidities and are not related to the surgical approach (28). The results of the present study also support the notion that comorbidities are a major risk factor contributing to mortality.

Interestingly, according to our findings, the in-hospital and 90-day mortality rates were substantially lower at 5.0% compared to the rates described in the literature (9). Three more death were observed due to the follow up period due to acute heart failure, thus the overall mortality increased at 15.0%. Notwithstanding, also the overall mortality rates of the present study are still comparable lower than the ones demonstrated in previous studies (9). One potential explanation might be that our patients were monitored after surgery at the ICU or an intermediate care ward and were not directly transferred to the regular ward. Therefore, the closer attention the frail patients received at the ICU may have permitted an early diagnosis and better management of any potential complications.

#### 4.2.2. Importance of ICU admission

According to our findings, the overall complication rates were relatively high at 50% with pneumonia being the most prevalent, followed by urinary tract infection, acute heart failure, and pleural effusion. Vaccaro et al. in their prospective study on older patients undergoing either surgery or conservative treatment for odontoid fractures reported a slight trend toward higher complication rates in the conservative group (30%) compared to that in the surgical group (36%) (30). Similar to our findings, pneumonia, respiratory problems, or dysphagia were quite frequently observed as adverse events post-surgery (30). Likewise, Charles et al. found an increased prevalence of complications with increasing age, and general medical complications such as respiratory and cardiac-related complications and delirium were most frequently noted irrespective of treatment type (29). One might argue that our complication rates were much higher than those reported by Vaccaro et al. and Charles et al. However, this disparity can be explained by the fact that our cohort constituted solely of patients aged 80 years and older; hence, our patients were more prone to experiencing adverse events attributable to their poor clinical profiles (CCI > 6). In agreement with our findings, Smith et al. found at least one major complication in 60% of the octogenarians undergoing surgery, with respiratory complications being the most prevalent (25). In the case of nonagenarians undergoing surgery for such fractures, the complication rate was relatively high (5/15, 33%), with respiratory complications being the most frequent (28). Due to the post-operative admission to the ICU, we were able to immediately detect clinical worsening and swiftly initiate therapy, such as antibiotic treatment in the case of pneumonia, preventing potential development of devastating infectious complications such as septic events. In a large Australian study on long term survival outcomes after ICU admission, older patients (mean age ≥70 years) had better survival rates even 9 years after surgery if they were admitted to an ICU post-surgery (31). According to a recent review and meta-analysis, older patients and especially octogenarians benefit from the post-operative admission to ICU, which contributes to lower morbidity and mortality rates, especially in emergency settings such as after emergency surgery (32). Although evidence is conflicting regarding the benefits of ICU admission after surgery in younger patients, we feel that the close monitoring of such debilitated subset of patients resulted in the low mortality rates presented here. Therefore, we hypothesize that, in case of emergency surgery of octogenarians, ICU therapy may lead to better outcomes, especially with respect to long term survival rates.

In the present study, fusion was achieved in all patients and the neurological status also improved significantly. Herein, it should be emphasized that the five reported deaths were caused by pneumonic sepsis, whereas surgical complications were not observed. The overall mortality rate was 15% over a 4-year follow-up period, and the deaths were not related to the surgery (cause of death: acute heart failure and malignant stroke). Given the dearth of robust evidence, no definitive conclusion can be drawn regarding the optimal therapy for odontoid fractures, especially in octogenarians. The largest study (165 operative and 157 conservative management patients) on this topic was conducted by AOSpine North America. This study provided level III evidence and advocated that surgical management should be favored, as conservative therapy conferred a higher risk of mortality when adjusting for confounding factors such as age and comorbidities (33). Furthermore, Vaccaro et al. suggested that operative management of such fractures leads to better functional outcomes, but this did not reach statistical significance (30). Kuntz et al. in their retrospective review on older patients (>65 years) found that conservative management results in higher failure rates with morbidity and mortality rates that were comparable to those associated with surgical management (34). In another study based on claim data and including 3,847 octogenarians, the study group reported that patients undergoing surgery did not show a higher incidence of in-hospital mortality, though higher rates of complications were observed (35).

#### 4.2.3. Surgical technique and intraoperative blood loss

The approach for the C1 lateral mass screws can be difficult, which is attributable to potential epidural bleeding from the venous plexus. This results in both a blockage in the surgeon’s view and difficulty in drill placement. Compared with the surgical techniques described in literature, the modified C1 technique presents with an advantage: after performing a midline approach and subperiosteal exposure of bony structures, the intraoperative CT scan allows for heightened accuracy of navigation-assisted techniques. The intraoperative insertion of reference markers in the surgical field allows for a quick registration by touching clearly defined landmarks, which can be repeated in less than 1 min in case of insecurity or movement due to instability. Anatomical landmarks or prolonged surface matching are not needed. However, potential hindrances can occur depending on the patient’s anatomy. For instance, in a prospective series, Yeom et al. described 51 Asian patients undergoing C1 fixation in a similar manner; however, anatomic considerations, such as a craniocaudal dimension of less than 4 mm of the C1 posterior arch, are most likely associated with cortical perforation (36). Furthermore, another difficulty of this technique is the presence of a smaller Atlas arch, a phenomenon usually seen in the Asian population (36). In addition, the presence of the ponticulus posticus, an ossification arch surrounding the dorsal course of the vertebral artery on the C1 posterior arch, endangers sagittal referencing from the superior boder of the posterior arch (37). Recognition of such anatomic variations via X-rays or CT imaging before surgery is pivotal in preventing tremendous complications during surgery. In the present study, no anatomical anomalies or variations were observed in preoperative imaging; thus, this technique could be applied without complications or damaging important structures such as the vertebral artery, the occipital nerve, as well as the C2 nerve root. Most importantly, bleeding from the epidural venous plexus can be avoided. By using an intraoperative navigation CT, we were able to track the patient’s anatomy in real time, thereby reducing errors and enhancing clinical outcomes.

Although we used a modified technique for the insertion of C1 pedicle screws as described by Tan et al. (38), we observed higher intraoperative blood loss with a mean of approximately 700 ml, which is much higher than described in previous studies (28, 39). One potential explanation may be that over 50.0% of our cases were taking anticoagulant agents, which may have contributed to higher intraoperative blood loss. Although administration of the agents was stopped before surgery, most of surgeries were performed in the acute setting; thus, the medications presumably still had an effect, as confirmed by the PTT results. Even if patients received antidotes before surgery, we still observed substantial intraoperative bleeding. This phenomenon may be attributable to the fact that octogenarians, due to their pure baseline history with multiple prolonged comorbidities, may require discontinuation intervals since their renal function is substantially impaired (creatinine clearance of less than 30 ml/min) (33). Impaired renal function will lead to prolonged effects of anticoagulant agents despite discontinuation before surgery or the use of antidotes to counter their effect (34, 35). We surmise that these two factors are inciting events resulting in higher intraoperative bleeding, despite the modified surgical technique associated to minimize intraoperative blood loss. Thus, a meticulous study of this debilitating cohort should be conducted preoperatively to prepare spine surgeons to sufficiently deal with such intraoperative complications.

#### 4.2.4. Conservative vs. surgical management

The optimal management of odontoid fractures type II, especially in octogenarians, remains a subject of debate. The union rates of type II odontoid fractures present the main factor for decision making and whether to initiate a conservative or surgical treatment approach. Previous studies suggest that non-surgical management with a cervical collar or halo vest are associated with high non-union rates of approximately >40% (28, 36), while surgical management with posterior screw fixation increases the fusion rates to >80% (28, 36). Gembruch et al., in their retrospective analysis of 125 patients with a mean age of 85.7 years sustaining a type II odontoid fractures, reported union rates of 70.0% in the conservative group, while a fusion rate of almost 91.0% was achieved in patients undergoing C1/C2 posterior screw fixation (37). Of note, the in-hospital and 90-day mortality did not differ significantly between both groups, while an increase was observed after 90 days, attributable to cardiopulmonary diseases and not directly to surgery. Considering these points, we concluded that surgical management seems to produce more beneficial outcomes for the elderly due to the high fusion rates, when compared to conservatively treated patients (37). In another retrospective study on conservative treatment among 58 older patients with type II odontoid fractures, Smith et al. reported high a mortality rate of 14% after 90 days of conservative treatment, while 64.0% required surgery due to substantial development of non-union fractures. Importantly, another 22.0% of the cases underwent surgery due to secondary non-union (38). In conjunction with the abovementioned studies, our patients also underwent surgery due to initial spinal instability. The major difference between our study and the aforementioned ones (37, 38) is that our patients presented with an acute trauma that caused the type II odontoid fracture; most importantly, a non-union displacement >4 mm, posterior displacement, as well as fracture coagulation of more than 10° were present. Hence, surgery was the only efficient approach to prevent further instability or even spinal cord injury due to instability at the atlantoaxial junction.

#### 4.2.5. Fusion rates after surgery or conservative management

In the current study, posterior screw fixation led to union rates of 100%. In concert with these findings, Issa et al., in their retrospective study on 15 nonagenarians who sustained acute odontoid type II fractures, reported fusion in all patients after C1-C2 pedicle screw fixation (28). Furthermore, Gembruch et al. also described higher fusion rates of 91.0% in patients undergoing surgery for type II odontoid fractures when compared to conservative management (37). Of note, the non-union rates after conservative management (over 30%) were high (37). Although, in accordance with our results and the aforementioned studies, surgical management seems to produce better outcomes for elderly patients, it should be noted that octogenarians are amenable to higher complications rates, especially after a surgical procedure, due to their poor baseline reserve. However, surgery seems to be inevitable when predisposing factors such as posterior displacement of the fracture, fracture distraction, lateral displacement on the posterior radiograph, and displacement greater than 4 mm (16) are present, as in the patients presented in the current study. It should be emphasized that posterior techniques are recommended in older patients since anterior screw fixation due to osteoporotic bones results in significantly lower fusion rates and is associated with higher morbidity and mortality rates (30). There is no clear consensus yet how to optimally treat such patients, but considering the results of the present study as well as of previous literature, posterior screw fixation unveils great rates of fusion which contributes to an improvement in the patient’s quality of life.

We believe that our data suggest that the decision on whether elderly patients should undergo surgery for a Type-II dens fracture should not be based on age alone, but on the value of regaining or preserving their quality of life. We found that while these patients presented with increased risks of morbidity and mortality, their neurological condition improved after surgery and most importantly, fusion could be achieved. A future prospective randomized trial is required in which elderly patients are allocated to specific arms of operative or non-operative management and are stratified by age. Such a study will help clarify the optimal therapeutic intervention for elderly patients with Type-II dens fractures.

### 4.3. Limitations

The main strength of the current study is that it is the first study to examine such a large cohort of octogenarians undergoing surgery for odontoid fractures. However, there are some limitations to this study. Selection bias could not be ruled out because of the retrospective study design. Additionally, as the data originated from a high-volume center, potential performance bias should also be considered. A longer follow-up period may uncover other relevant information that was not captured in the current study. Functional outcomes could not be sufficiently reconstructed using patients’ medical records; hence, they were omitted from this analysis. However, we believe that the impact of surgery could be adequately evaluated using the ASIA grading system. Our study employs a modified Goel-Harms technique, which, while rooted in the traditional approach, introduces specific variations. These modifications were consistently applied but may introduce outcomes slightly different from the conventional technique. However, the clinical significance of such nuances, given the foundational consistency with the original method, is expected to be minimal. Since patients suffered from an acute trauma and the admission to the hospital was less than 24 h after the initial trauma, a delay in the diagnosis was absent. All CT images showed the abovementioned parameters, as evaluated by experienced neuroradiologists and spine surgeons (KK, BI). Since octogenarians carry the high risk of perioperative morbidity and mortality, an interdisciplinary discussion was held with experienced anesthesiologists to evaluate the potential risks associated with surgery. However, since the enrolled patients suffered from unstable fractures which are unlikely to achieve a bony union by treatment by a halo vast and can presumably also result in progressive myelopathy and contribute to spinal cord injury due to instability at the atlantoaxial junction (16, 40, 41), a surgical procedure via posterior screw fixation was recommended, while a conservative treatment approach was considered as insufficient and associated with undue risks for progression of spinal instability. Finally, a lack of a conservatively treated control group is a major limitation of this study.

## 5. Conclusion

With the global trend of increasing life expectancy due to accelerating improvements in the quality of healthcare worldwide, spine surgeons are frequently confronted with the management of odontoid fractures in the geriatric population. The complication and mortality rates of such fractures in octogenarians are relatively high. However, bone union and preservation of the neurological status are also major goals of the therapeutic strategies. Despite an often high comorbidity rate, we still recommend that surgery should be considered in patients over 80 years of age. A variety of approaches should be evaluated by healthcare professionals when treating elderly frail patients. Regardless, a clear discussion with the patient and relatives regarding the potential risk is unambiguously recommended.

## Data availability statement

The original contributions presented in this study are included in the article/supplementary material, further inquiries can be directed to the corresponding author.

## Author contributions

BI and PL performed material preparation and data collection and analysis. PL wrote the first draft of the manuscript. GG, KK, AU, and BI commented on previous versions of the manuscript. All authors contributed to the study conception and design, read, and approved the final manuscript.
